# Introduction of an *N*-Glycosylation Site into UDP-Glucuronosyltransferase 2B3 Alters Its Sensitivity to Cytochrome P450 3A1-Dependent Modulation

**DOI:** 10.3389/fphar.2016.00427

**Published:** 2016-11-14

**Authors:** Tatsuro Nakamura, Naho Yamaguchi, Yuu Miyauchi, Tomoki Takeda, Yasushi Yamazoe, Kiyoshi Nagata, Peter I. Mackenzie, Hideyuki Yamada, Yuji Ishii

**Affiliations:** ^1^Laboratory of Molecular Life Sciences, Graduate School of Pharmaceutical Sciences, Kyushu UniversityFukuoka, Japan; ^2^The Cabinet Office, Government of JapanTokyo, Japan; ^3^Department of Environmental Health Science, Tohoku Medical and Pharmaceutical UniversitySendai, Japan; ^4^Department of Clinical Pharmacology, Flinders University, AdelaideSA, Australia

**Keywords:** UDP-glucuronosyltransferase, UGT, cytochrome P450, P450, CYP, protein–protein interaction

## Abstract

Our previous studies have demonstrated functional protein–protein interactions between cytochrome P450 (CYP) 3A and UDP-glucuronosyltransferase (UGT). However, the role of carbohydrate chains of UGTs in the interaction with CYP is not well understood. To address this issue, we examined whether CYP3A1 modulates the function of UGT2B3 which lacks potential glycosylation sites. We also examined whether the introduction of *N-*glycosylation to UGT2B3 affects CYP3A-dependent modulation of UGT function. To introduce a potential glycosylation site into UGT2B3, Ser 316 of UGT2B3 was substituted with Asn by site-directed mutagenesis. A baculovirus-Sf-9 cell system for expressing CYP3A1 and UGT2B3/UGT2B3(S316N) was established using a Bac-to-Bac system. Glycosylation of UGT2B3(S316N) was demonstrated in this expression system. The microsomal activity of recombinant UGT was determined using 4-methylumbelliferone as a substrate. The effect of CYP3A1 co-expression on UGT function was examined by comparing the kinetic profiles between single (UGT alone) and double expression (UGT plus CYP) systems. The kinetics of the two expression systems fitted a Michaelis-Menten equation. When the 4-MU concentration was varied, co-expression of CYP3A1 lowered the *V*_max_ of UGT2B3-mediated conjugation. Conversely, for UGT2B3(S316N), the *V*_max_ in the dual expression system was higher than that in the single expression system. The data obtained demonstrate that the introduction of *N-*glycosylation to UGT2B3 alters its sensitivity to CYP3A1-dependent modulation while CYP3A1 enhanced UGT2B3(S316N) activity, and wild-type UGT2B3 was suppressed by CYP3A1. These data suggest that *N-*glycosylation of UGT is one of the determinants regulating the interaction between CYP3A and UGT.

## Introduction

The potential for drug metabolism varies from one individual to another. Therefore, drug metabolism capacity is a factor determining whether a drug produces pharmacological or adverse effects. Drug metabolism is classified into phase 1 and phase 2 reactions. The major reaction of phase 1 is oxidation in which cytochrome P450 (CYP) plays a key role (Guengerich and Rendic, 2010). Of all the enzymes including phase 2, UDP-glucuronosyltransferase (UGT) is mediating conjugation with glucuronic acid supplied from a cofactor (UDP-glucuronic acid, UDPGA) (Rowland et al., 2013). CYP and UGT are bound to the endoplasmic reticulum membrane, and their catalytic domains are localized in the cytosolic and luminal sides, respectively (Shepherd et al., 1989; Yamazaki et al., 1993). These enzymes have long been considered to work separately. However, we have shown that several UGTs can be trapped by a CYP1A1-immobilized affinity column (Taura et al., 2000). Moreover, our previous studies have shown that interaction between CYP3A4 and UGT2B7 alters the regio-selectivity of UGT2B7-catalyzed morphine glucuronidation (Takeda et al., 2005, 2009). Furthermore, CYP3A4 alters the function of UGT1A subfamily isoforms in an isoform and allelic variant specific fashion (Ishii et al., 2014). Rat UGTs are efficiently co-immunoprecipitated with anti-CYP3A antibody and the CYP3A-UGT complex is catalytically active for 4-methylumbelliferone glucuronidation (Ishii et al., 2007). Although the mechanisms of the interaction between CYP3A and UGT have not been clarified, the J-helix region of CYP3A4 is a candidate region involved in the interaction with UGT2B7 (Takeda et al., 2009). The domain(s) of UGT2B7 involved in the interaction with CYP3A4 has also been reported (Miyauchi et al., 2015). It is suggested that the hydrophobic regions at both the carboxyl terminal and luminal anchoring segment of UGT2B7 are crucial. However, further details about the interaction remain to be clarified.

Many UGTs have consensus sequences for *N-*glycosylation (Mackenzie, 1990a,b; Barbier et al., 2000; Nakajima et al., 2010; Nagaoka et al., 2012). The sequence can be generalized as NX(S/T), where X is any amino acid except proline, and approximately 70-90% of this sequence is glycosylated at its asparagine (N) residue (Gavel and von Heijne, 1990). Earlier studies have reported that *N-*glycosylation affects the enzyme activity of UGTs (Mackenzie, 1990a,b; Barbier et al., 2000; Nakajima et al., 2010; Nagaoka et al., 2012). We have reported that CYP3A4 interacts with UGT1A1, 1A6, 1A7, and 2B7 (Takeda et al., 2005; Ishii et al., 2014). All of these UGT isoforms possess potential *N-*glycosylation sites. However, the role of *N-*glycosylation of UGT in the interaction with CYP3A is unknown. Although the inhibition of *N-*glycosylation reduces UGT1A9 activity, deglycosylation of the mature form (*N-*glycosylated form) did not affect its catalytic properties (Nakajima et al., 2010). Thus, *N-*glycosylation has been suggested to be important for the protein folding of UGT. However, multiple mutations of the three *N-*glycosylation sites on UGT2B7 have different effects on substrate specificities (Nagaoka et al., 2012). Because of the important roles of *N-*glycosylation in UGT folding and function, we hypothesized that the *N-*glycosylation of UGT affects the interaction between CYP3A and UGT. To address this issue, we focused on rat UGT2B3 (Mackenzie, 1987) which does not possess potential glycosylation sites. We examined whether CYP3A1 modulates UGT2B3 and whether the introduction of *N-*glycosylation to UGT2B3 affects the CYP3A-dependent modulation of the UGT function.

## Materials and Methods

### Materials

4-Methylumbelliferone (4-MU) and alamethicin were purchased from Sigma-Aldrich (St. Louis, MO, USA). UDP-Glucuronic acid (UDPGA) trisodium salt and 4-MU-β-D-glucuronide were obtained from Nakalai Tesque (Kyoto, Japan). Endoglycosidase H (EndoH) was purchased from New England Biolabos (Beverly, MA, USA). All other reagents were of the highest grade commercially available.

### Animals

Animal experiments in this study were conducted following the approval of the Ethics Committee for Animal Experiments of Kyushu University. Male Wistar rats (7 weeks-old) were obtained from Charles River Japan (Tokyo, Japan) and were maintained for one week with free access to water and a suitable diet under a 7 a.m. to 7 p.m. light/dark cycle. For isolation of total RNA, liver tissue was quickly cut into small fragments, immediately immersed in liquid nitrogen, and stored at -80°C until required.

### Expression System

Baculovirus for expressing CYP3A1 and UGT2B3 in Sf-9 cells was prepared using a Bac-to-Bac system (Invitrogen). CYP3A1 cDNA was subcloned from P91023(B) (Nagata et al., 1999) into pFastBac1 vector at an *EcoR*I site. The sequencing reaction was carried out using a Big Dye^®^ Terminator v3.1 Cycle Sequencing Kit (Life Technologies). Then, cDNA sequences were confirmed by an ABI 3130xl Genetic Analyzer. CYP3A1 has two synonymous and one non-synonymous nucleotide substitutions: C1035G, G1074A, and T1055A, respectively, when compared with the database (Gonzalez et al., 1985; GenBank accession M10161). T1055A causes an amino acid substitution, M352K, and this CYP3A1 is known to be catalytically active (Nagata et al., 1999). It was used as the CYP3A1 in this study.

UGT2B3 cDNA was amplified from the total RNA of Wistar rat liver by a reverse transcription-polymerase chain reaction (RT-PCR), and cloned into pFastBac1. Isolation of total RNA and the RT-reaction were carried out by a method previously reported (Mutoh et al., 2006). To amplify the cDNA, nested PCR with two primer sets was used. The primer set for the first round PCR was UGT2B3(-20,-1)F1, 5′- TAA GGA TTT TGA TTT TTA AG-3′ and UGT2B3(1647,1628)R1, 5′-CAT AAA T TA GAA TGA GGC TG-3′. The primer set for the second round PCR was PstI-UGT2B3(-5,15)F2, 5′-AAC TGC AGT TAA GAT GCC TGG GAA GTG G-3′, and PstI-UGT2B3(1615, 1596)R2, 5′-AAC TGC AGT GTA GTG CAT TGT AA ATG AG-3′ with restriction sites (*Pst*I) underlined. The PCR was carried out with LA-Taq DNA polymerase (TaKaRa Bio, Kyoto) using the manufacturer’s recommended protocol with the following components: 1xLA-Taq buffer, 2 mM MgCl_2_, 2.5 mM each dNTP, the primers F1 and R1 (5 μM each) and the cDNA in 100 μL (first round PCR). The amplification was carried out using the PROGRAM TEMP CONTROL SYSTEM PC-800 (ASTEC, Fukuoka, Japan) with 94°C, 4 min-(94°C, 1 min, 50°C, 1 min, 72°C, 2 min)×30 cycles-72°C, 20 min –4°C, ∞. The second round PCR was carried out as above but using primers F2 and R2. The resulting PCR products were restricted with PstI and cloned into pFastBac1. The sequence was confirmed by DNA sequencing as described above. UGT2B3 has a non-synonymous nucleotide substitution, T1498A, which causes a single amino-acid change, S500T. We used this as a wild-type UGT2B3 in this study.

*Escherichia coli* DH10Bac was transformed either with pFastBac1 plasmid carrying UGT2B3 or CYP3A1 cDNA to prepare recombinant bacmid DNA. Production and amplification of recombinant baculovirus and expression of recombinant proteins in Sf-9 cells were carried out according to the method described previously (Ishii et al., 2014).

### The Introduction of a Potential *N-*Glycosylation Site into UGT2B3

UGT2B3 exhibits 83% identity to UGT2B2 (Mackenzie, 1986) in amino acid sequences. The Asn 316 of UGT2B2 is a potential site for glycosylation. Therefore, the Ser 316 of UGT2B3 was replaced with Asn by site-directed mutagenesis (SDM), and an expression system for the UGT2B3(S316N) mutant was constructed in a similar way as described above. The primers for the SDM were designed by Quick Change Primer Design (Agilent Technology). The primers used were UGT2B3(931, 964)(947G→A)SDM-F, 5′-GGG TCA ATG GTC AGC AAC ATG ACA GAA GAA AAG G-3′ and UGT2B3(964, 931)(947C→T)SDM-R, 5′-CCT TTT CTT CTG TCA TGT TGC TGA CCA TTG ACC C-3′. The procedures were carried out according to the manufacturer’s recommendations. The introduction of the mutation at the appropriate position and the absence of other unwanted mutations were confirmed by DNA sequencing.

### Kinetic Analysis

The kinetic analysis was performed using 100 μg microsomal protein. The amount of microsomal protein used was unified by adding control baculosomes. The activity of UGT2B3-catalyzed glucuronidation was determined by high-performance liquid chromatography (HPLC) with 4-MU as a substrate (Hanioka et al., 2001). The microsomes and alamethicin were mixed and preincubated for 30 min on ice. The assay was started by adding UDPGA and incubation was performed for 60 min at 37°C. The reaction was stopped with 100 μL 1 M trichloroacetic acid (TCA). After chilling on ice for 30 min, the incubation mixture was centrifuged (15,000 rpm, 4°C, 10 min). The supernatant containing the 4-MU glucuronide formed was analyzed by HPLC with a fluorescence detector (Ex 315 nm, Em 375 nm).

### Data Analysis

The kinetics fitted a Michaelis-Menten equation, and the kinetic parameters were calculated using GraphPad Prism software (GraphPad Software Inc., San Diego, CA, USA). The statistical difference in kinetic parameters between UGT2B3 single expression and UGT2B3-CYP3A1 dual expression was evaluated by repeating the extra sum-of-squares *F* test.

### Immunoblotting

Proteins were determined by the method of Lowry et al. (1951) with bovine serum albumin as a standard. SDS-polyacrylamide gel electrophoresis (SDS-PAGE) was performed according to Laemmli (1970). Proteins separated by SDS-PAGE were electroblotted to a polyviniliden difluoride membrane. UGT2B3 and UGT2B3(S316N) were detected by a goat anti-mouse low-pI form UGT antibody (Mackenzie et al., 1984). CYP3A1 was detected by rabbit anti-CYP3A2 antibody (Nagata et al., 1990). Immunochemical detection was conducted either with horseradish peroxidase (HRP)-conjugated secondary antibodies, HRP-rabbit anti-goat IgG (MP Biomedicals, Santa Ana, CA, USA), or HRP-donkey anti-rabbit IgG (GE Healthcare, Piscataway, NJ, USA). These were diluted 10,000- and 40,000-fold before use, respectively. Clarity Western ECL Substrate (Bio-Rad, Hercules, CA, USA) was used as the substrate of HRP, and the chemiluminescence emitted was analyzed by a ChemiDoc MP System (Bio-Rad).

### His-Tag Pull-Down Assay

Introduction of hemagglutinin (HA)-tag at the carboxyl terminus of UGT2B3 was carried out by a two-step PCR. At the first step, primer HA(21,1)-UGT2B3(1590,1573)R, 5′-ATC TGG AAC ATC GTA TGG GTA CTC ATT CTT CAT TTT CTT-3′ and primer F2 were used. The PCR reaction was basically the same as that above except that pFastBac1-UGT2B3 was the template. In the second step, PstI-TCA-HA(27,1)R, 5′-AAC TGC AGT CAA GGG TAA TCT GGA ACA TCG TAT GGG TA-3′ and primer F2 were used (Underline, *Pst*I site). In the second step, the PCR products of the first step were used as a template. The PCR products were restricted with PstI and cloned into pFastBac1. To construct HA-tagged UGT2B3(S316N), an SDM described above was carried out with pFastBac1-UGT2B3-HA. To construct hexa-histidine (His)_6_-tagged CYP3A1, PCR was carried out with the following primers: NotI-CYP3A1(-4,16)F, 5′-ATA AGA ATG CGG CCG CAG GGA TGG ACC TGC TTT CAG-3′ and XhoI-CYP3A1-Histag(1510,1494)R, 5′-CCG CTC GAG TCA GTG ATG GTG ATG GTG ATG TGA TCC AGT TAT GAT TTC A-3′ (Underlines: *Not*I and *Xho*I sites, respectively). The PCR products were purified and restricted with *Not*I and *Xho*I and then subcloned into pFastBac1 restricted with the same enzymes. Their recombinant baculovirus was prepared as described above. Sf-9 cells were transfected with the recombinant virus for either CYP3A1-(His)_6_ and/or UGT2B3-HA/UGT2B3(S316N). The resulting microsomes were solubilized with sodium cholate and the pulled down assays were carried out as described previously (Miyauchi et al., 2015).

### Modeling of the Structure of the UGTs

The models were constructed using Phyre^2^ (Protein Homology/Analogy Recognition Engine V 2.0) web server, http://www.sbg.bio.ic.ac.uk/phyre2/html/ (Kelley et al., 2015) with UGT2B7 (amino acid residue from 285 to 450) as a template (Miley et al., 2007). Although the program is designed for prediction using multiple templates, only UGT2B7 was selected as a template in this case.

## Results

### Expression of UGT2B3 and the Glycosylation Mutant, UGT2B3(S316N)

**FIGURE 1:**
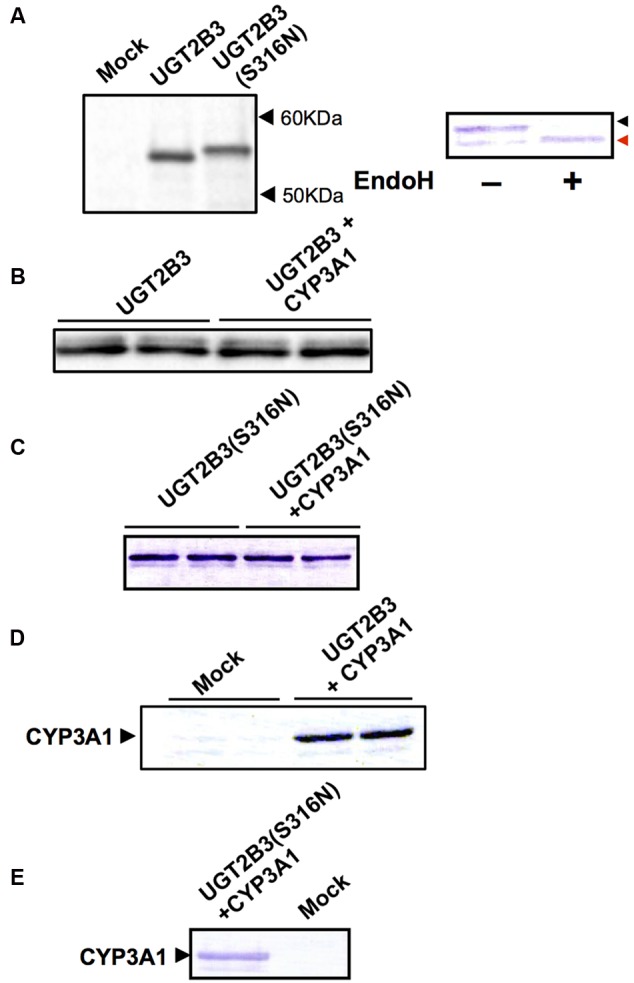
**Immunoblots of the expression of UGT2B3, UGT2B3(S316N), and CYP3A1 in Sf-9 cells.** To obtain microsomes simultaneously expressing CYP3A1 and UGT2B3/UGT2B3(S316N), Sf-9 cells were transfected with recombinant baculovirus for CYP3A1 and UGT2B3/UGT2B3(S316N). The lanes labeled “UGT2B3” and “UGT2B3(S316N)” show microsomal samples of single transfected cells. The lanes labeled “UGT2B3+CYP3A1” and “UGT2B3(S316N)+CYP3A1” show double transfected cells. Baculosomes (5 μg protein) from UGT2B3 single-expressing Sf-9 cells were electrophoresed (SDS-PAGE). For baculosomes of CYP3A1-UGT2B3 co-expression systems, protein amounts equivalent to UGT2B3 for the single-expressing system were used. Similarly, baculosomes (3 μg protein) from UGT2B3(S316N) single-expressing Sf-9 cells were electrophoresed. For baculosomes of CYP3A1-UGT2B3(S316N) co-expression systems, protein amounts equivalent to UGT2B3(S316N) for the single-expressing system were used. Mock represents the baculosomes (10 μg protein) from Sf-9 cells infected with baculovirus without passenger DNA which served as controls. The proteins in the gel were electrically transferred to a polyvinilidene difluoride membrane, and blotted with goat anti-mouse low p*I* form UGT **(A-C)** and rabbit anti-CYP3A2 **(D,E)**, antibodies, respectively. In right panel of **(A)**, baculosomes (25 μg protein) from UGT2B3(S316N)-expressing Sf-9 cells were treated with Endoglycosidase H (EndoH, 500 U) at 37°C for 17 h. Four microgram protein of the digest was subjected to immunoblotting with goat anti-mouse low p*I* form UGT antibody as a primary antibody. Black and red arrow heads represent original and deglycosylated UGT2B3(S316N), respectively.

UGT2B3 was expressed in Sf-9 cells, and the protein band in the 50-60 kDa molecular mass range that was immunoreactive toward anti-mouse low pI form UGT antibody and was absent in control microsomes was judged to be UGT2B3 (**Figure 1A**). To introduce an *N-*glycosylated sugar chain to UGT2B3, we referred to UGT2B2 (Mackenzie, 1986) which has a potential *N-*glycosylation site and exhibits high similarity to UGT2B3 in primary sequence. Then, the Ser316 of UGT2B3 was substituted to Asn to establish an expression system for UGT2B3(S316N). The immune-reactive band with higher molecular mass and absent in control microsomes was judged to be UGT2B3(S316N)(**Figure 1A**). Thus, the molecular mass was increased by the introduction of a potential glycosylation site suggesting newly introduced *N-*glycosylation. This was also supported by a reduction in size after EndoH-treatment (**Figure 1A**).

### Effect of Co-Expression of CYP3A1 on UGT2B3- and UGT2B3(S316N)-Catalyzed Glucuronidation

**FIGURE 2:**
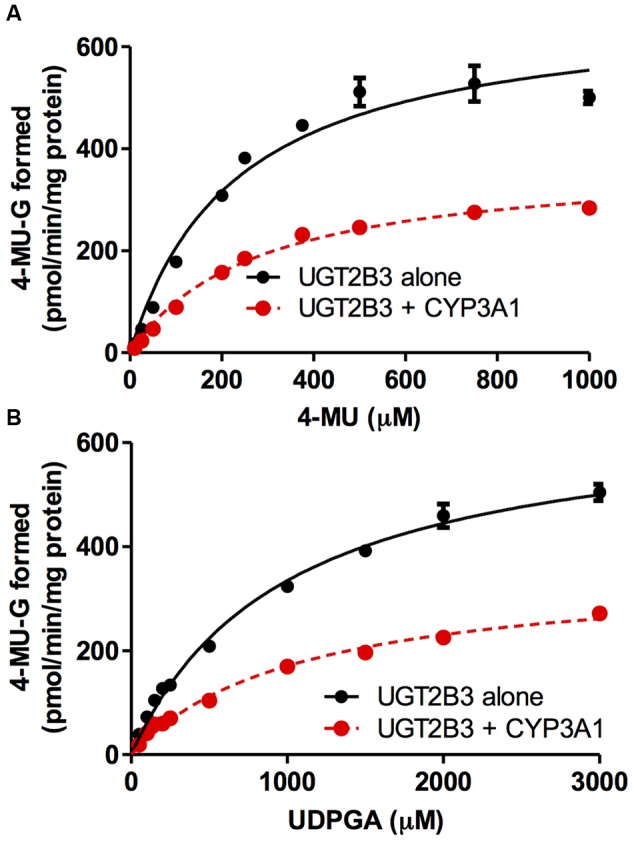
**Michaelis-Menten plots of 4-MU glucuronidation catalyzed by UGT2B3 in the absence and presence of CYP3A1 co-expression.** The plots of UGT2B3 single- and UGT2B3-CYP3A1-dual are shown. The kinetics obtained by varying the 4-MU concentration **(A)** and UDPGA concentration **(B)** are shown. In **(A)**, the 4-MU concentration was varied over the range 10-1000 μM while the UDPGA concentration was fixed at 2 mM. In **(B)**, the UDPGA concentration was varied over the range 50 μM–3 mM while the 4-MU concentration was fixed at 1 mM. The total amount of protein added to the assay mixture was standardized at 100 μg. For this, when necessary, control baculosomes were added to the reaction mixture. Kinetic parameters were calculated by fitting the curve to a Michaelis-Menten equation, and they are listed in **Table 1**.

**FIGURE 3:**
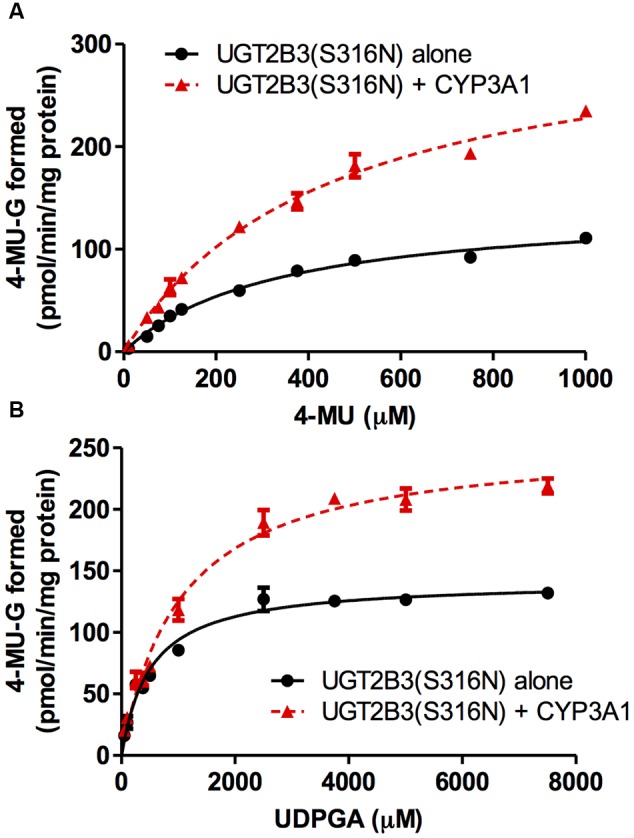
**Michaelis-Menten plots of 4-MU glucuronidation catalyzed by UGT2B3(S316N) in the absence and presence of CYP3A1 co-expression.** The plots for UGT2B3(S316N) single- and UGT2B3(S316N)-CYP3A1-dual are shown. The kinetics obtained by varying the 4-MU concentration **(A)** and UDPGA concentration **(B)** are shown. In **(A)**, the 4-MU concentration was varied over the range 10-1000 μM while the UDPGA concentration was fixed at 2 mM. In **(B)**, the UDPGA concentration was varied over the range 50 μM–7.5 mM while the 4-MU concentration was fixed at 1 mM. The total amount of protein added to the assay mixture was standardized at 100 μg. For this, when necessary, control baculosomes were added to the reaction mixture. The kinetic parameters were calculated by fitting the curve to a Michaelis-Menten equation, and they are listed in **Table 2**.

CYP3A1 was co-expressed in Sf-9 cells with UGT2B3 or UGT2B3(S316N). Sf-9 cells, the microsomes of which express UGT2B3 or UGT2B3(S316N) having a comparable band intensity to that in the single expression system, were selected and subjected to further investigation of their enzymatic properties (**Figures 1B–E**). The amount of UGT2B3 and microsomal protein used for the assay was also unified between the single and dual expression systems. For this, we rendered the protein level uniform with baculosomes obtained from Sf-9 cells infected with control baculovirus. When CYP3A1 was expressed together with UGT2B3, the *V*_max_ of UGT2B3-catalyzed 4-MU glucuronidation was significantly decreased (**Figure 2**; **Table 1**). The kinetic profiles for both the single and double expression could be fitted to a Michaelis-Menten equation. When the 4-MU concentration was varied, the *V*_max_ was reduced significantly while the *K*_m_ was comparable (**Table 1**). Furthermore, the same was also true for the kinetics by varying the UDPGA concentration (**Figure 3**; **Table 1**). The *V*_max_ was lowered by CYP3A1 cotransfection while the *K*_m_ for UDPGA was comparable. Therefore, CYP3A1 suppressed the activity of UGT2B3 which lacks potential glycosylation sites. However, when CYP3A1 was expressed together with UGT2B3(S316N), the *V*_max_ of UGT2B3(S316N)-catalyzed 4-MU glucuronidation was significantly increased (**Figure 3**; **Table 2**). The kinetic profiles for both single and double expression could be fitted to a Michaelis-Menten equation. When the 4-MU concentration was varied, the *V*_max_ was increased significantly while the *K*_m_ was comparable (**Table 2**). Both the *V*_max_ and *K*_m_ of UGT2B3(S316N)-catalyzed 4-MU glucuronidation was significantly increased by varying the UDPGA concentration (**Figure 3**; **Table 2**). In sharp contrast to the wild-type UGT2B3, co-expression of CYP3A1 increased UGT2B3(S316N) activity.

**Table 1:** Kinetic parameters for 4-MU glucuronide formation catalyzed by Sf-9 microsomes expressing UGT2B3 alone and UGT2B3 + CYP3A1: kinetics by varying 4-MU or UDPGA concentration.

	*K*_m_ (μM)	*V*_max_ (pmol/min/mg protein)	CLint (μg/min/mg protein)
By varying 4-MU concentration			
UGT2B3	231 ± 31	682 ± 33	2.95
UGT2B3+CYP3A1	278 ± 20	377 ± 11^∗^	1.36
By varying UDPGA concentration			
UGT2B3	982 ± 70	664 ± 20	0.676
UGT2B3+CYP3A1	1088 ± 103	356 ± 14^∗^	0.327

*Data were fitted to a Michaelis-Menten equation (**Figure 2**). Results are the estimated value ± SE. ^∗^Significantly different from UGT2B3 single expression (*p* < 0.0001).*

**Table 2:** Kinetic parameters for 4-MU glucuronide formation catalyzed by Sf-9 microsomes expressing UGT2B3(S316N) alone and UGT2B3(S316N) + CYP3A1: kinetics by varying 4-MU or UDPGA concentration.

	*K*_m_ (μM)	*V*_max_ (pmol/min/mg protein)	CLint (μg/min/mg protein)
By varying 4-MU concentration			
UGT2B3(S316N)	330 ± 33	143 ± 6	0.433
UGT2B3(S316N)+CYP3A1	444 ± 43	329 ± 43^∗^	0.740
By varying UDPGA concentration			
UGT2B3(S316N)	513 ± 51	142 ± 4	0.277
UGT2B3(S316N)+CYP3A1	1040 ± 100^∗^	256 ± 8^∗^	0.245

*Data were fitted to a Michaelis-Menten equation (**Figure 3**). Results are the estimated value ± SE. ^∗^Significantly different from UGT2B3(S316N) single expression (*p* < 0.0001).*

### Effect of EndoH-Treatment on the Activity of UGT2B3(S316N)

**FIGURE 4:**
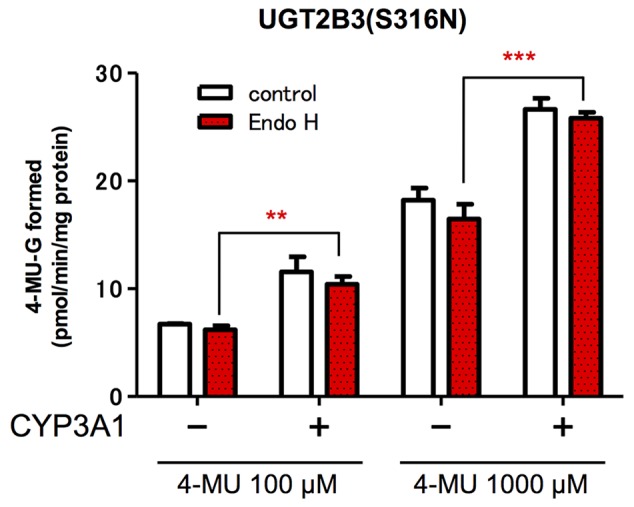
**Effect of EndoH-treatment on the glucuronidation catalyzed by UGT2B3(S316N).** Baculosomes (20 μg protein) from UGT2B3(S316N) single- and UGT2B3(S316N)-CYP3A1 dual-expressing Sf-9 cells were treated with Endoglycosidase H (EndoH, 250 U) at 37°C for 1 h. The UGT activity was assayed with 100 or 1000 μM 4-MU and UDPGA fixed at 3 mM. Significantly different from UGT2B3(S316N) single expression (^∗∗^*p* < 0.001; ^∗∗∗^*p* < 0.0001).

The glucuronidation activities of microsomes expressing UGT alone, UGT2B3(S316N), and UGT and CYP, UGT2B3(S316N)-CYP3A1, were compared with or without EndoH-treatment (**Figure 4**). The 4-MU glucuronidation activity in UGT2B3(S316N)-CYP3A1 was significantly higher than the UGT2B3(S316N) single expression even after treatment with EndoH. The deglycosylation did not affect the activity of UGT2B3(S316N).

### Protein–Protein Interaction of CYP3A1 and UGT2B3/UGT2B3(S316N)

**FIGURE 5:**
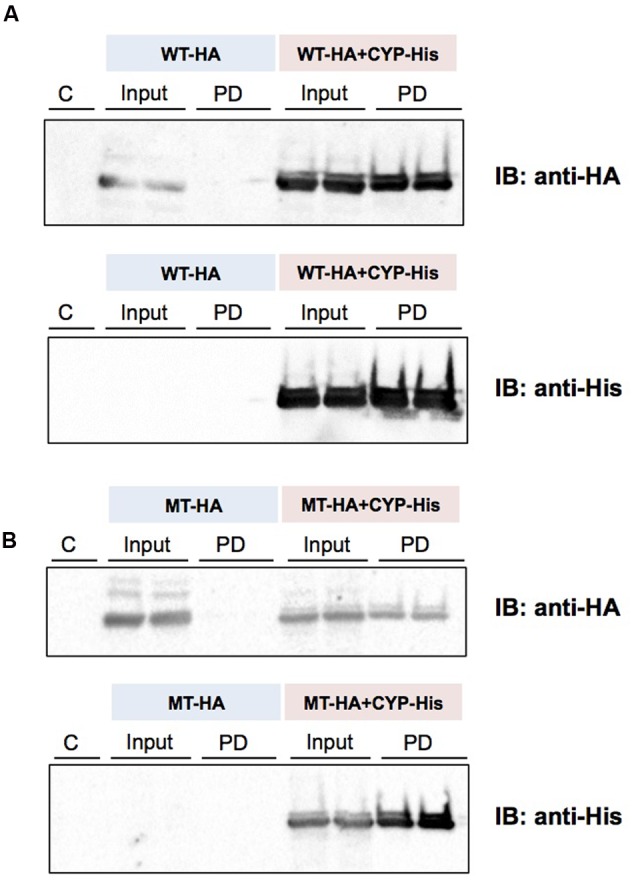
**Detection of an interaction between CYP3A1 and UGT2B3/UGT2B3(S316N) by His-tag pull-down assay.** UGT2B3-HA and UGT2B3(S316N)-HA were expressed in the presence and absence of coexpressed His-CYP3A1. The pull-down assay was performed according to methods previously published (Miyauchi et al., 2015) with slight modification. Microsomes (2 mg protein/mL) were solubilized with sodium cholate. Then the solubilized microsomes were used in each assay. His-CYP3A1 and proteins trapped by this P450 were eluted with buffer containing imidazole at a high concentration. Each protein was detected by immunoblotting with a specific antibody: rabbit anti-HA or rabbit anti-His. Solubilized microsomes and His-CYP3A1–trapped samples are indicated as Input (30% of the input) and PD (pull down), respectively. In **(A)**, HA-tagged wild-type UGT2B3 (WT-HA) was used. In **(B)**, HA-tagged UGT2B3(S316N) (MT-HA) was used. WT-HA+CYP-His and MT-HA+CYP-His indicate microsomes coexpressing UGT2B3-HA or UGT2B3(S316N)-HA with His-CYP3A1, respectively. Duplicate assays were carried out. For each pull-down sample, one half of the precipitate was subjected to SDS-PAGE for detection with either HA or His while the lanes shown by “C” represent a negative control sample obtained by the same procedures but using solubilized control microsomes as an input, with microsomes prepared from Sf-9 cells transfected with control baculovirus alone. Details are described in the Section “Materials and Methods”.

(His)_6_-tagged CYP3A1 was constructed and, the baculovirus encoding it subsequently co-transfected with either UGT2B3 or UGT2B3(S316N) which were tagged with HA. A band immunoreactive to an anti-HA antibody was observed in the precipitates (**Figure 5**). However, there was no pull down when they were expressed singly. Therefore, both HA-tagged UGT2B3 and UGT2B3(S316N) were pulled down by (His)_6_-tagged CYP3A1 which suggests that CYP3A1 interacts with not only UGT2B3 but also UGT2B3(S316N).

### Prediction of the Structure of the Carboxyl Terminal Domain of Wild-Type UGT2B3 and a Glycosylated Mutant UGT2B3(S316N)

**FIGURE 6:**
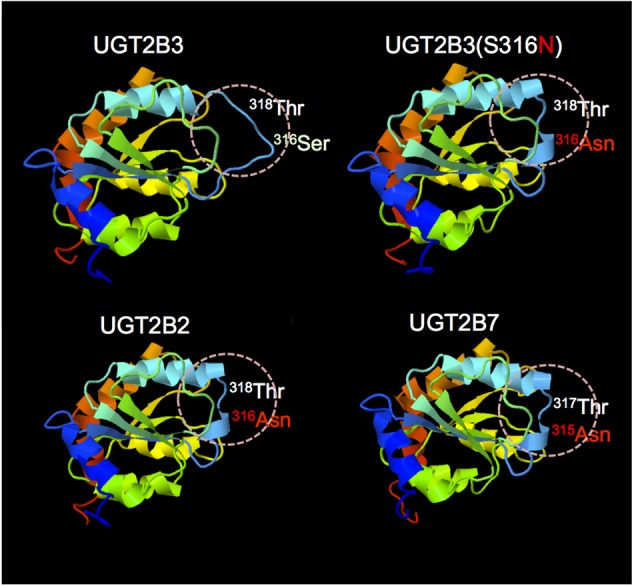
**Comparison of the predicted structure of UGT2B3 wild-type, UGT2B3(S316N), UGT2B2, and UGT2B7: the region spanning from 285 to 450.** The models were constructed using the Phyre2 web server (Kelley et al., 2015) with the UGT2B7 (amino acid residue from 285 to 450) as a template. Template: UGT2B7, Miley et al. (2007) (DOI: 10.2210/pdb2o6l/pdb).

The structures of part of the carboxyl terminal domain of UGT2B3 and UGT2B3(S316N) were predicted by *Phyre^2^* with UGT2B7 as template (**Figure 6**). For comparison, the structure of UGT2B3 which has Asn at 316 as a potential *N-*glycosylation site was also predicted. For reference, the prediction was also carried out for the UGT2B7 and UGT2B2 with UGT2B7 as a template. In UGT2B7, Asn 315 is the corresponding residue. Overall, it seems that the predicted structures are very similar to each other. The structure around residue 316 or 315 (for UGT2B7), a β-strand structure was observed except for UGT2B3. Although the prediction does not include the effect of the glycosylation chain, it is assumed that substitution of Ser 316 for Asn altered the partial structure of UGT2B3.

## Discussion

The effect of CYP3A1 on UGT2B3 which lacks a potential glycosylation site was studied. Since it is evident that CYP3A1 modulates UGT2B3 activity, *N-*glycosylation is not essential for CYP3A1 to modulate this UGT. Furthermore, this study demonstrates that the introduction of *N-*glycosylation to UGT2B3 alters UGT sensitivity to a functional protein–protein interaction with CYP3A: wild-type UGT2B3 which lacks*N-*glycosylation sites is suppressed by CYP3A1, whereas this P450 increases UGT2B3(S316N) activity. These results suggest that although the *N-*glycosylation of UGT is not essential for modulation by CYP3A, the *N-*sugar chain linked to UGT is one of the factors regulating this interaction between CYP3A and UGT. In this study, we focused on the effect of CYP3A1 on UGT function. To allow a comparison, we carried out several transfection experiments to obtain microsomes for UGT single and CYP3A1-UGT dual microsomes with a comparable UGT level and the results shown in **Tables 1** and **2** allow an examination of the effect of CYP3A1 on each UGT. Although the activity of UGT2B3 and UGT2B3(S316N) cannot be simply compared between **Tables 1** and **2**, it seems that the introduction of *N-*glycosylation to UGT2B3 at this position reduces the glucuronidation activity. Based on this, it is likely that CYP3A1 increases the function of the glycosylated mutant UGT2B3(S316N) to approach that of the wild type. In general, the methods that involve introduction of mutation(s) to the potential glycosyation site to inhibit glycosylation are a well-known strategy to investigate the role of glycosylation on enzyme function. In some enzymes, the activity was increased by reducing the glycosylation site, while in some other enzymes, the activity was decreased (Skropeta, 2009). *N-*Glycosylation is involved in the protein folding of UGT1A9 and important for catalytic function (Nakajima et al., 2010). However, deglycosylation of the mature UGT1A9 did not affect its function. In the case of UGT2B3(S316N), *N-*glycosylation resulted in positive modulation by CYP3A1 (**Figure 4**). However, the increase in UGT activity was consistent even after EndoH-treatment (**Figure 4**). Therefore, it is suggested that the engineered glycosylated chain of UGT2B3(S316N) is no longer required after maturation and fixing the conformation of UGT2B3(S316N). This was also true for UGT2B3(S316N) without co-expression of CYP3A1. Taken together, these findings suggest that the complex formation with CYP3A1 affects the conformation of UGT2B3(S316N). Although the kinetics were not studied when examining the effect of deglycosylation, the activity was consistent at both low and high substrate concentrations. It is assumed that deglycosylation did not alter the affinity to the substrate. Since glycosylation of UGT has been shown to be important during synthesis of the enzyme but not once the mature enzyme is formed (Nakajima et al., 2010), it is assumed that the CYP3A1-dependent modulation of UGT2B3(S316N) that we observed is due to the interaction of CYP3A4 with the secondary structure of UGT2B3(S316N) during the process of formation of the mature UGT enzyme. However, whether glycosylation or merely the presence of the Asn at that site is sufficient to alter structure and function, remains to be clarified in a future study. Nevertheless, our current study suggests that the introduction of a potential glycosylation site to UGT2B3 alters its CYP3A1-dependent modulation.

UGT2B7 is known to interact with CYP3A4 (Takeda et al., 2005, 2009; Miyauchi et al., 2015). Furthermore, the crystal structure of the cofactor-binding domain of UGT2B7 (from residues 285 to 450) has been determined (Miley et al., 2007). So far, that is the sole example of a crystal structure of mammalian UGTs. Concerning the structural differences between UGT2B3 and UGT2B3(S316N), the structures of the corresponding region were predicted by *Phyre^2^* with UGT2B7 as a template (**Figure 6**). The results were very similar except around the 316th residue. So, on the basis of the prediction, the substitution of Ser at the 316th residue of UGT2B3 by Asn converted the random coil to a β-strand structure (**Figure 6**). It is reasonable to suppose that the combination of the structural difference and the *N-*glycosylation in UGT2B3(S316N) resulted in a different susceptibility to modulation by CYP3A1.

This study suggests that *N-*glycosylation of UGT is one of the determinants of the functional interaction between CYP3A and UGT. UGT function is known to be affected by *N-*glycosylation (Mackenzie, 1990a,b; Barbier et al., 2000; Nakajima et al., 2010; Nagaoka et al., 2012). Many UGTs have *N-*glycosylation at more than two positions (Barbier et al., 2000; Nagaoka et al., 2012). Therefore, the role of *N-*glycosylation of the other UGTs is very important for functional protein–protein interactions with CYP3A. Further studies are necessary to determine the importance of *N-*glycosylation of UGTs in the UGT-CYP interactions.

## Author Contributions

Participated in research design: TN, NY, YM, TT, YY, KN, PM, HY, and YI. Conducted experiments: TN, NY, and YI. Contributed new reagents or analytical tools: TN and NH. Performed data analysis: TN, NY, and YI. Wrote or contributed the writing of the manuscript: TN, NY, YM, and YI.

## Conflict of Interest Statement

The authors declare that the research was conducted in the absence of any commercial or financial relationships that could be construed as a potential conflict of interest.
